# Inverse scheduling method for aircraft flat-tail assembly production based on improved genetic algorithm

**DOI:** 10.1038/s41598-025-23898-9

**Published:** 2025-11-17

**Authors:** Tengda Li, Min Hua, Junliang Wang, Wei Qin

**Affiliations:** 1 Shanghai Jiao Tong University, USC-SJTU Institute of Cultural and Creative Industry, Shanghai, 200240 China; 2 Department of Industrial Energy Products, China Mobile Shanghai Industrial Research Institute, Shanghai, 201206 China; 3 Institute of Artificial Intelligence, Donghua University, Shanghai, 201620 China; 4 Department of Industrial Engineering and Management, Shanghai Jiao Tong University, Shanghai, 200240 China

**Keywords:** Flat-tail assembly production, Self-adaptive driving mechanism, Inverse scheduling, Improved genetic algorithm, Engineering, Mathematics and computing

## Abstract

The manufacturing process of the aircraft flat-tail assembly is complex and discrete. It typically involves manual assembly at fixed stations with variable shift teams. However, uncertainties can arise even after a scheduling scheme is created, leading to non-optimal or even infeasible schedules. To address this issue, a new scheduling strategy called ‘inverse scheduling’ has been proposed by incorporating the concept of inverse optimization. Notably, this is the first application of inverse scheduling in the complex manufacturing process of aircraft flat-tail assembly. This paper presents a multi-objective optimization model for the inverse scheduling problem of flat-tail assembly production. The scheduling objectives include minimizing the maximum delay penalty cost and minimizing the assembly time adjustment cost. To address the limitations of traditional mathematical planning methods in terms of efficiency and solution quality, an improved genetic algorithm is proposed. This algorithm combines the genetic algorithm with a local search strategy to solve the large-scale inverse scheduling problem. Additionally, an inverse scheduling strategy based on the self-adaptive tolerance-driving mechanism is designed to enhance the algorithm’s efficiency and effectively handle order delay exception events. The effectiveness of the self-adaptive tolerance driving mechanism and the inverse scheduling method is verified through case studies in enterprises. Comparative analysis demonstrates that the proposed method significantly outperforms traditional rescheduling strategies by avoiding high sequence adjustment and material handling costs, offering a more practical and efficient solution for managing disruptions in complex assembly systems.

## Introduction

Aircraft flat-tail is a discrete manufacturing process for complex aviation products. Its assembly process involves multiple assembly frames, multiple components, and mixed-flow assembly of various configuration products. Aircraft flat-tail assembly is a typical discrete assembly. Its assembly process has the following characteristics: (1) multiple assembly processes, the aircraft flat-tail passes through each assembly process in turn, which is a flow assembly operation; (2) multiple parallel assembly frames, each assembly process corresponds to an assembly station, and each assembly station has at least one assembly frame; (3) multi-configuration flat-tail mixed flow assembly, the same type of aircraft flat-tail has multiple configurations, and the assembly process of each configuration is the same, but there are differences in the assembly cycle time. Therefore, aircraft flat-tail assembly production scheduling is a typical flexible flow-shop scheduling problem (FFSP). FFSP has been extensively studied for decades^1–8^ and has great significance for actual production^9,10^.

At the same time, the aircraft flat-tail assembly adopts the manual assembly production mode of the fixed station and variable shift team, thus during the assembly process, abnormal events such as deviations in assembly time often occur, and the accumulation of these deviations in the assembly process may result in the assembly production delay of orders, or even lead to delayed delivery of orders, which brings high delay penalties and lower customer satisfaction, which can affect the credit rating of the aircraft manufacturing company. A schedule obtained in the FFSP may be optimal before production begins; however, real manufacturing systems do not consistently meet ideal conditions, and the parameters may change, making the original schedule non-optimal or even infeasible. To improve the market competitiveness of the aircraft manufacturing company and enhance the on-time delivery capability of flat-tail orders, the assembly process of the aircraft flat-tail must be effectively managed, and abnormal events in the assembly schedule should be handled in a timely manner.

Extensive work has been done on dynamic scheduling methods under production exceptions, and the most commonly adopted method at present is rescheduling. Rescheduling refers to readjusting the production sequence of each processing task according to specific scheduling rules when an abnormal production event causes the pre-scheduled plan to deteriorate in performance or become infeasible, ensuring the production plan can be executed smoothly. The most common rescheduling methods include fully reactive rescheduling, predictive rescheduling, and pre-reactive rescheduling. The rescheduling method is widely used in manufacturing systems where the production scheduling sequence can be dynamically and flexibly adjusted. However, for aircraft flat-tail assembly, since the rescheduling of the flat-tail production sequence will lead to high flat-tail handling costs and components distribution costs, the long transfer time of the flat-tail between assembly frames, as well as the corresponding adjustment of the parts processing plan and the procurement plan of outsourced parts, is required. Therefore, the rescheduling method is not suitable for coping with the dynamic abnormal events of flat-tail assembly production.

Unlike rescheduling, inverse scheduling (IS) is a scheduling method that does not alter the pre-scheduling sequence. It enables the manufacturing system to address dynamic abnormal events that occur during the production process by adjusting specific scheduling parameters of the pre-scheduling scheme, thereby ensuring the optimized performance of the manufacturing system. For aircraft flat-tail assembly, when an abnormal assembly event occurs, the assembly time of each process can be adjusted indirectly by reasonably adjusting the number of workers at each assembly station. Thus, the method of inverse scheduling has higher practical operability.

As a novel direction in the field of production scheduling, the inverse scheduling problem has attracted the interest of many scholars. Brucker^11^ studied a two-machine shop floor inverse scheduling problem with the minimization of the maximum completion time as the scheduling objective, investigated the sufficient conditions for the existence of the optimal solution of the inverse scheduling when the production sequence of machining tasks on the two machines is different, and proved that even if all the production sequences of machining tasks on the two machines are the same, the two machine shop floor inverse scheduling problem is still an NP-hard problem. Pham^12^ studied the parallel machine inverse scheduling problem, with the objective of minimizing total completion time, and proved that there are sufficient conditions for the optimal solution of the parallel machine inverse scheduling problem. Chen et al.^13^ investigated the single-machine inverse scheduling problem, aiming to minimize the total completion time. Yang et al.^14^ studied two types of inverse batch scheduling problems: one is an inverse batch scheduling problem with bounded Hamming distance weights, and the other is a partial inverse batch scheduling problem that considers both free and fixed positions. Zhang et al.^15^ studied the inverse scheduling problem by minimizing the total weighted completion time as the scheduling objective, with processing time and objective function weights as the scheduling adjustment parameters, and the improvement of the objective function values before and after the inverse scheduling, and measured the adjustment magnitude of the scheduling parameters using three different paradigms of 1, 2, and ∞, respectively, and verified them with an example of the berth allocation problem at terminals in the shipbuilding industry. Mou et al.^16^ investigated an improved genetic algorithm to solve the single-machine inverse scheduling problem, with the objective of minimizing the weighted total completion time. They proposed an improved genetic algorithm incorporating a local search strategy. To enhance the performance of the improved genetic algorithm, the authors developed an effective encoding mechanism, a fitness value evaluation mechanism, a feasible solution initialization mechanism, and a local search mechanism. The local search mechanism is particularly effective in improving the solution search capability. Zhao^17^ studied the welding shop inverse scheduling problem (WSISP), considering energy consumption, and established a single objective model to minimize changes in processing parameters. A multi-objective model was established to minimize changes in processing parameters, minimize total energy consumption, and minimize maximum completion time. Based on the original Grey Wolf Optimizer (GWO) algorithm, the Improved Grey Wolf Optimizer (IGWO) algorithm and the Multi-Objective Grey Wolf Optimizer (MOGWO) algorithm were designed to solve single- and multi-objective WSISP problems. Wu et al.^18^ investigated the inverse scheduling for the final assembly of a turbine in a changing environment, considering uncertainty factors during the assembly process and worker and crane constraints. The authors proposed a multi-project inverse scheduling model to minimize total worker cost. An algorithm combining particle swarm optimization (PSO) to assign workers and Tabu search (TS) to create an overtime plan was adopted to solve the problem. Yim et al.^19^ considered an inverse interval scheduling problem on a single machine to reduce the non-preemptive job intervals with the least cost. The inverse interval scheduling problem has been proven to be strongly NP-hard, and a polynomial-time approximation scheme has been designed to solve it. Wang et al.^20^ researched a new Welding Inverse Scheduling Problem (WSISP) and proposed an IGWO algorithm with variable neighbourhood search to solve the WSISP. Togo et al.^21^ presented two methods for estimating the weighting factors of the objective function in the scheduling problem using historical data, given information on operation times and setup costs. The author then proposed a machine learning-based method and an inverse optimization-based method that utilize the input/output data of the scheduling problems when the weighting factors of the objective function are unknown. These two methods were applied to a multi-objective parallel machine scheduling problem and a real-world chemical batch plant scheduling problem. They were proven to be effective in solving both problems. Mou et al.^22^ proposed an energy-efficient distributed permutation flow-shop inverse scheduling problem to minimize adjustment and energy consumption simultaneously. An effective hybrid collaborative algorithm with a cooperative search scheme was designed to solve the problem, and the heuristic and random methods were improved to initialize the population. A double-population cooperative search link based on a learning mechanism was designed to balance the algorithm’s global exploration and local development capabilities. To solve the inverse job-shop scheduling problem (JSP), Wang et al.^23^ proposed a hybrid solution based on a genetic algorithm (GA) and improved particle swarm optimization (PSO) to minimize parameter adjustments. The solution was presented as block coding with a decimal mechanism, allowing both processes and parameters to be optimized simultaneously. Alexander et al.^24^ considered the single-machine scheduling problem with given release dates and the objective to minimize the maximum penalty. A dual and an inverse problem were introduced and proven to be solvable in polynomial time. The optimal function value of a sub-problem in the dual problem of a branch-and-bound algorithm was proposed for the original single-machine scheduling problem. Zhang et al.^25^ investigated the multi-mode, multi-project inverse scheduling problem in the turbine assembly workshop under uncertain conditions. A modified integer and categorical particle swarm optimization algorithm combined with Tabu search (MICPSO-TS) was proposed to address this issue. Wu et al.^26^ introduced a hybrid multi-objective evolutionary algorithm based on decomposition and particle swarm optimization for solving flexible job shop inverse scheduling problems (FJISP). Recent advances in related domains, such as the multi-objective metaheuristic for maintenance planning^27^ and the modelling of disassembly line balancing^28^, further enrich the algorithmic toolkit available for complex scheduling problems.

The following characteristics of the current research on inverse scheduling can be observed from a review of existing studies on inverse scheduling: (1) numerous studies have been conducted on inverse scheduling for single machines, two machines, and parallel machines, but few studies have focused on complex scheduling systems. (2) The focus of research on inverse scheduling is mainly on theoretical research, including the proof of sufficient conditions for optimal solutions of inverse scheduling and the selection of metrics for the adjustment range of scheduling parameters. At the same time, there is a lack of research on the application of inverse scheduling to practical engineering problems. (3) At present, the solution method of inverse scheduling is mainly a mathematical planning method, and there is no efficient method for solving large-scale inverse scheduling problems. (4) In terms of modelling of inverse scheduling, most scholars only adopt the method of mixed integer programming for small-scale problems of single and two machines, and there are few modelling methods for large-scale complex scheduling problems, such as flexible flow shop scheduling problems, especially for aircraft flat-tail assembly production. Summary of related work on inverse scheduling and our contributions can be seen in Table 1.

**Table 1:** Summary of related work on inverse scheduling and our contributions.

Reference	Problem type	Methodology	Limitations/Focus	Our contribution
Brucker^11^	Two-machine shop	Theoretical Analysis	Sufficient conditions, NP-hardness proof	Application-focused: Addresses large-scale FFSP in a real-world context.
Mou et al.^16^	Single-machine	Improved GA	Small-scale, theoretical problem	Algorithm: Hybrid GA with adaptive local search for large-scale problems.
Zhao^17^	Welding shop	Improved GWO	Energy consumption focus	Model: Multi-objective model for assembly time & cost in aviation.
Wu et al.^18^	Turbine assembly	PSO-TS	Worker and crane constraints	Mechanism: Novel adaptive tolerance-driven triggering mechanism.
Wang et al.^23^	Job-shop	GA-PSO Hybrid	Minimizing parameter adjustments	Validation: Comprehensive vs. rescheduling & other metaheuristics.
Zhang et al.^25^	Turbine (Multi-mode)	MICPSO-TS	Uncertainty under changeable environment	Generality: Framework applicable to other complex products (ships, turbines).
This study	FFSP for Aircraft Flat-tail	Adaptive IGA + Tolerance Mechanism	Practical application, stability, cost-effectiveness	Solves all above limitations

Based on the current research status and the identified gaps, the main contributions of this paper are fourfold:

1. A novel multi-objective inverse scheduling model was formulated for the Flexible Flow-Shop Scheduling Problem (FFISP) in aircraft flat-tail assembly.
2. An adaptive tolerance-driven mechanism was proposed that dynamically triggers inverse scheduling based on real-time system performance (self-adjustment ability) and remaining adjustment capacity, preventing both excessive reactivity and delayed responses.
3. A hybrid Improved Genetic Algorithm (IGA) that effectively combines the global search capability of GA with a problem-specific local search strategy was developed to solve the large-scale NP-hard FFISP efficiently.
4. We comprehensively demonstrate the superiority of the proposed method through comparisons with traditional rescheduling, other driving mechanisms, and state-of-the-art algorithms, validated by both numerical experiments and a real-world industrial case study.

## Inverse scheduling model for flat-tail assembly production

### Problem description

The aircraft flat-tail assembly shop is a flexible flow shop, and the flat-tail assembly production inverse scheduling problem is the flexible flow-shop inverse scheduling problem (FFISP), which can be described as follows: *m* assembly processes, and *m*≥2; each assembly process has *x*_*k*_ assembly frames (*x*_*k*_≥1), and at least one assembly process *x*_*k*_≥2; *n* assembly tasks {*J*_*1*_,*J*_*2*_,...,*J*_*n*_ }, each assembly task belongs to different configurations of the flat-tail of a specific type of aircraft, and its assembly process is the same, each assembly task needs to go through all the assembly processes in turn, and for the assembly process with multiple assembly frames, each assembly process can choose any available frame, the assembly time of each process on each assembly frame is determined by the number of workers, the more workers there are, the shorter the corresponding assembly time, the number of workers on each assembly frame has an upper limit, and the assembly time of each assembly process is a discrete value in a specific closed interval; the scheduling sequence of each assembly task is known, that is, the assignment scheme of each assembly frame and the sequential assembly order of each assembly task is predetermined and cannot be changed.

The scheduling task of FFISP is: when an abnormal event of order delay occurs while ensuring that the scheduling sequence determined by the original production scheduling plan remains unchanged, a reasonable inverse scheduling strategy is adopted to indirectly adjust the assembly time of each assembly station by reasonably adjusting the number of workers operating at each assembly station (increasing or decreasing) and adopting the smallest possible worker adjustment cost, to ensure that the order delay penalty cost minimal.

The FFISP problem is a bi-objective optimisation problem. On the one hand, the FFISP problem should minimise the total delay penalty cost of each assembly task, and on the other hand, the FFISP problem should minimise the operator adjustment cost. These two optimisation objectives are contradictory. By adding more operators at each assembly station, it is clear that the assembly speed of each station can be increased to a greater extent, which in turn shortens the assembly cycle of the flat-tail and reduces the total delay penalty cost of all assembly tasks. Still, at the same time, more operators also incur higher human resources costs. Therefore, it is necessary to model and study the multi-objective optimisation problem of FFISP.

In order to facilitate the establishment of the FFISP inverse scheduling model, the following assumptions are made in the scheduling model of this paper under the condition that the actual characteristics of the flat-tail assembly are guaranteed to the greatest extent: (1) the assembly time of each process is a discrete value with a definite closed interval; (2) once each assembly process starts, no interruption is allowed in the middle; (3) there is no assembly sequence constraint between the processes of different assembly tasks, and the sequence constraints of assembly processes need to be considered between different processes of the same assembly task; (4) each assembly frame can only assemble one aircraft flat-tail at the same time; (5) each aircraft flat-tail can only be assembled on one assembly frame at the same time; (6) the handling time of the aircraft flat-tail between different frames is neglected; (7) the rated work efficiency of assembly workers is the same and remains constant, and the cost of workers is mainly determined by the assembly hours.

### Mathematical model

#### Notations and variables

*I,e* -- Index of the assembly frames, *i,e* =*1,2,…,m*.

*j,k* -- Index of the assembly tasks, *j,k* = *1,2,…,n*.

*n*_*i*_ -- the total number of processes for the assembly task *j*.

*h,l* -- assembly process set, *h* = *1,2,…,n*_*j*_.

*M*_*i*_ -- the *i*^*th*^ assembly frame.

*J*_*j*_ -- the *j*^*th*^ assembly task, (*j = 1,2,…,n*), *n* is the total number of assembly tasks.

*c*_*i*_ -- scheduling sequence on the *i*^*th*^ assembly frame.

*c* -- scheduling sequence of assembly tasks on the *1~m* assembly frame, *c* = (*c*_*1*_*,c*_*2*_*,…,c*_*m*_)

*wn*_*i*_ -- the sequence of the number of worker assignments corresponding to the scheduling sequence *c*_*i*_ on the *i*^*th*^ assembly frame.

*wn* -- the sequence of the number of worker assignments corresponding to the scheduling sequence *c* on the *1~m* assembly frames.

$$\begin{array}{c}\approx \\ {wn}_{i}\end{array}$$ -- the sequence of the number of worker assignments corresponding to the scheduling sequence *c*_*i*_ on the *i*^*th*^ assembly frame after the adjustment of the number of workers.

$$\begin{array}{c}\approx \\ wn\end{array}$$ -- the sequence of the number of worker assignments corresponding to the scheduling sequence *c* on the *1~m* assembly frames after the adjustment of the number of workers.

*W*_*max*_ – the maximum number of assembly workers on all assembly frames, that is, the total number of assembly workers.

*t*_*i*_ -- the assembly time series corresponding to the sequence of the number of worker assignments on the *i*^*th*^ assembly frame.

*t* -- the assembly time series corresponding to the sequence of the number of worker assignments on the *1~m* assembly frames.

$$\begin{array}{c}\approx \\ {t}_{i}\end{array}$$ -- the assembly time series corresponding to the sequence of the number of worker assignments on the *i*^*th*^ assembly frame after the adjustment of the number of workers.

$$\begin{array}{c}\approx \\ t\end{array}$$ -- the assembly time series corresponding to the sequence of the number of worker assignments on the *1~m* assembly frame after the adjustment of the number of workers, and $$\begin{array}{c}\approx \\ t\end{array}=(\begin{array}{c}\approx \\ {t}_{1}\end{array},\begin{array}{c}\approx \\ {t}_{2}\end{array},\dots ,\begin{array}{c}\approx \\ {t}_{m}\end{array})$$.

*M*_*jh*_ -- the optional assembly frame set for the *h*^*th*^ process of the assembly task *j*.

*n*_*jh*_ -- the number of candidate assembly frames for the *h*^*th*^ process of the assembly task *j*.

*O*_*jh*_ -- the *h*^*th*^ process of the assembly task *j*.

*M*_*ijh*_ -- the *h*^*th*^ process of the assembly task *j* on the *i*^*th*^ assembly frame.

*wnum*_*imax*_ -- the upper limit of the number of assembly workers on the *i*^*th*^ assembly frame.

*wn*_*ijh*_ -- the number of assembly workers on *i*^*th*^ the assembly frame for the *h*^*th*^ process of the assembly task *j*.

$$\begin{array}{c}\approx \\ {wn}_{ijh}\end{array}$$ -- the number of assembly workers on *i*^*th*^ the assembly frame for the *h*^*th*^ process of the assembly task *j* after the adjustment of the number of workers.

*T*_*ijh*_ -- the rated working hours for the *h*^*th*^ process of the assembly task *j* on the *i*^*th*^ assembly frame.

*P*_*ijh*_ -- the assembly time candidate set for the *h*^*th*^ process of the assembly task *j* on the *i*^*th*^ assembly frame, ${P}_{ijh}\in [{P}_{ijh}^{-},{P}_{ijh}^{+}]$, ${P}_{ijh}^{-}$ and ${P}_{ijh}^{+}$ are the lower and upper bounds of the assembly time, respectively, and are determined by the number of assembly workers.

*p*_*ijh*_ -- the assembly time for the *h*^*th*^ process of the assembly task *j* on the *i*^*th*^ assembly frame, ${p}_{ijh}=\frac{{T}_{ijh}}{{wn}_{ijh}}$, and ${p}_{ijh}\in {P}_{ijh}$.

$$\begin{array}{c}\approx \\ {p}_{ijh}\end{array}$$ -- the assembly time for the *h*^*th*^ process of the assembly task *j* on the *i*^*th*^ assembly frame after the adjustment of the number of workers, $$\begin{array}{c}\approx \\ {p}_{ijh}\end{array}=\frac{{T}_{ijh}}{\begin{array}{c}\approx \\ {wn}_{ijh}\end{array}}$$, and $$\begin{array}{c}\approx \\ {p}_{ijh}\end{array}\in {P}_{ijh}$$.

*s*_*jh*_ -- the start time of the *h*^*th*^ process of the assembly task *j*.

$$\begin{array}{c}\approx \\ {s}_{jh}\end{array}$$ -- the start time of the *h*^*th*^ process of the assembly task *j* after the adjustment of the number of workers.

*c*_*jh*_ -- the finish time of the *h*^*th*^ process of the assembly task *j*.

$$\begin{array}{c}\approx \\ {s}_{jh}\end{array}$$ -- the finish time of the *h*^*th*^ process of the assembly task *j* after the adjustment of the number of workers.

*C*_*j*_ – the assembly completion time for the *j*^*th*^ assembly task.

*D*_*j*_ – the delivery time for the *j*^*th*^ assembly task.

$$\begin{array}{c}\approx \\ {C}_{j}\end{array}$$ -- the assembly completion time for the *j*^*th*^ assembly task after the adjustment of the number of workers.

*cw* -- the wage cost per unit of time for assembly workers.

*cp*_*j*_ -- the delay penalty cost per unit time for the assembly task *j*.

*X*_*ijh*_ – 0,1 variable, which takes the value 1 if the process *O*_*jh*_ is processed on *i*^*th*^ the assembly frame.

*X*_*ijhkl*_ – 0,1 variable, which takes the value 1 if the process *O*_*ijh*_ is processed on *i*^*th*^ the assembly frame before the process *O*_*ikl*_.

#### Objective function and constraints

1 $$f_{1} = \min \sum\limits_{i = 1}^{I} {p_{i} } \cdot \max \{ C_{i} - D_{i} ,0\}$$

2 $$f_{2} = \min \sum\limits_{i = 1}^{m} {\sum\limits_{j = 1}^{n} {\sum\limits_{h = 1}^{{n_{j} }} c } } \tilde{w} \cdot \mathop {p_{ijh} }\limits^{ \approx } \cdot \max \left\{ {0,\left( {w\tilde{n}_{ijh} - wn_{ijh} } \right)} \right\}$$

3 $$\mathop s\nolimits_{jh} + \mathop X\nolimits_{ijh} \cdot \mathop p\nolimits_{jh} \le \mathop c\nolimits_{jh} \& \mathop {\mathop s\nolimits_{jh} }\limits^{ \approx } + \mathop X\nolimits_{ijh} \cdot \mathop {\mathop p\nolimits_{jh} }\limits^{ \approx } \le \mathop c\nolimits_{jh}$$

4 $$c_{jh} \le s_{j(h + 1)} {\text{ \& }}c_{jh}^{ \approx } \le s_{j(h + 1)}^{ \approx }$$

5 $$FT_{{i{\kern 1pt} j_{1} x}} \cdot W_{j1j2} \le ST_{{i{\kern 1pt} j_{2} x}} \cdot W_{j1j2} ,{\kern 1pt} \forall i,x.\forall j_{1} ,{\kern 1pt} j_{2} \in j$$

6 $$c_{jh} \le s_{j(h + 1)} {\text{ \& }}\mathop {c_{jh} }\limits^{ \approx } \le \mathop {s_{j(h + 1)} }\limits^{ \approx }$$

7 $$\mathop s\nolimits_{jh} + \mathop X\nolimits_{ijh} \cdot \mathop p\nolimits_{jh} \le \mathop c\nolimits_{jh} \& \mathop {\mathop s\nolimits_{jh} }\limits^{ \approx } + \mathop X\nolimits_{ijh} \cdot \mathop {\mathop p\nolimits_{jh} }\limits^{ \approx } \le \mathop c\nolimits_{jh}$$

8 $$P_{ijh} \approx \in [P_{ijh}^{ - } ,...,P_{ijh}^{ + } ]$$

9 $$FT_{{i{\kern 1pt} j_{1} {\kern 1pt} x}} \cdot W_{{j1{\kern 1pt} j2}} \le ST_{{i{\kern 1pt} j_{2} {\kern 1pt} x}} \cdot W_{{j1{\kern 1pt} j2}} ,\forall i,x.\forall j_{1} ,j_{2} \in j$$

The Eq. (1) and Eq. (2) are the two objective functions of the FFISP problem, the Eq. (1) represents minimizing the maximum delay penalty cost, and the Eq. (2) represents minimizing the assembly time adjustment cost.

The Eq. (3) to Eq. (9) are the constraints of the FFISP problem. The Eq. (3) and Eq. (4) indicate the order of completion of each process of each assembly task, with the later process being later than the earlier process; the Eq. (5) and Eq. (6) indicate that the assembly of at most one aircraft flat-tail can be carried out simultaneously at the same time on the same assembly frame; the Eq. (7) indicates the process constraint of each assembly task, that is, the same process of each component can be assembled on at most one assembly frame at the same time. The Eq. (8) indicates the assembly time adjustment constraint, that is, the adjustment range of the assembly time cannot exceed the carrying capacity of each assembly station for the assembly workers, and can only be adjusted within the specified range; the Eq. (9) indicates that the number of assembly workers on each assembly frame cannot be greater than the maximum number allowed for that assembly frame.

### Mathematical model formulation and exact solution

To provide a benchmark for small-scale instances and validate the correctness of the proposed model, we reformulate the FFISP as a Mixed-Integer Programming (MIP) model suitable for exact solvers like GUROBI or CPLEX. The model uses the notations defined previously.

#### Objective functions

The bi-objective problem is transformed into a single objective using a weighted sum method, where α is a weighting coefficient between 0 and 1.

#### Minimize

10 $$F = \alpha \cdot \mathop {max}\nolimits_{j} (\mathop {cp}\nolimits_{j} \cdot \max (0,\mathop {\mathop C\nolimits_{j} }\limits^{ \approx } - \mathop D\nolimits_{j} )) + (1 - \alpha ) \cdot cw \cdot \sum\nolimits_{i} {\sum\nolimits_{j} {\sum\nolimits_{h} {\left| {\mathop {\mathop {wn}\nolimits_{ijh} }\limits^{ \approx } - \mathop {wn}\nolimits_{ijh} } \right|} } } \cdot \mathop {\mathop p\nolimits_{ijh} }\limits^{ \approx }$$

#### Subject to

Constraints (3) - (9) from the original model, linearized where necessary.

The max function in the first objective is linearized using standard techniques (introducing auxiliary variables and constraints).

The absolute value in the second objective is linearized by introducing two non-negative variables.

We implemented this MIP model in Python using the GUROBI 10.0.1 solver. A small-scale instance (Experimental group 1: 6 tasks × 4 processes) was solved to optimality. The computation was performed on a PC with an AMD Ryzen 7 6800H processor and 32 GB RAM. The solver found the optimal solution with an objective value *F* = 36.8 (with α = 0.5) in approximately 30 seconds.

This result confirms the model’s correctness. However, the exponential growth in computation time for larger instances (e.g., the 12x4 case was terminated after 5 minutes without reaching proven optimality) underscores the NP-hard nature of the FFISP and justifies the necessity of developing efficient metaheuristics like the IGA for practical, large-scale problems.

## Inverse scheduling driving mechanism

For the inverse scheduling problem of order delay exception events caused by assembly progress anomalies, a suitable inverse scheduling driving mechanism needs to be selected that can quickly respond to the delay exception events while maintaining good scheduling performance. The everyday inverse scheduling driving mechanisms are cycle-driven, event-driven, and hybrid-driven. The event-driven mechanism is widely used because it can respond to abnormal events in the production process in real-time. However, event-driven scheduling may lead to frequent adjustments, which can affect the stability of assembly production. Therefore, it is necessary to establish a buffer mechanism for inverse scheduling driving and filter out unnecessary inverse scheduling. For this reason, many scholars have developed specific dynamic scheduling driving methods tailored to the characteristics of their respective problems. Liu et al.^29^ proposed an event-driven rescheduling mechanism based on the profit-and-loss model, while Song^30^ proposed a driving mechanism based on the tolerance for delivery date deviation. Although these improved event-driven rescheduling strategies mitigate frequent rescheduling due to dynamic abnormal events to some extent, they do not consider the impact on manufacturing system performance. They cannot adopt a more appropriate driving mechanism based on the performance of the manufacturing system and the execution of the production schedule.

For flat-tail assembly: (1) due to the characteristics of manual work of assembly workers, assembly work hours are often a value that fluctuates in a particular range, and the deviation of assembly work hours in a specific period may be compensated as the assembly progresses. Hence, the deviation in assembly work hours has varying degrees of impact on assembly progress and order delivery delays at different stages of the assembly process. It is necessary to set different inverse schedules for each assembly stage. (2) At the same time, there is a big difference in the adjustable capacity of the production plan in different assembly stages. At the early stage of assembly, the adjustment space of the assembly plan is larger, and as the execution of the assembly plan progresses, the adjustment space of the assembly plan becomes smaller and smaller, therefore, the inverse scheduling driving mechanism for flat-tail assembly should also consider the influence of the adjustable space in different assembly stages.

For different assembly stages, the self-adjustment ability of the assembly system for delivery delay exceptions is called the self-adjustment performance indicator δ_1_, and the assembly production plan of the assembly system’s own adjustment space is called the adjustment space indicator δ_2_. The self-adjustment ability indicator is related to the completion of the assembly progress, the less the assembly completion process, the greater the self-adjustment ability, δ_1_ as shown in Eq. (11); the adjustment space indicator is related to the adjustable range of the assembly time of the remaining assembly process and the length of the order delay, so δ_2_ is defined as the Eq. (12), the larger of δ_2_, the larger of the adjustment space.

11 $$\delta_{1} = \frac{{C_{{{\mathrm{Done}}}} }}{{C_{{T{\mathrm{otal}}}} }}$$

12 $$\delta_{2} = |\frac{{C_{{\text{Adjustable time}}} - C_{{\text{Delay time}}} }}{{C_{{\text{Adjustable time}}} }}|$$

**Fig. 1.**
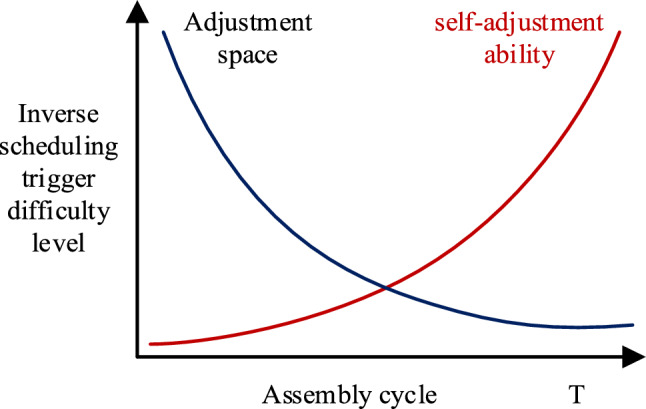
Self-adjustment ability indicator and adjustment space indicator.

The self-adjustment ability indicator δ_1_ and the adjustment space indicator δ_2_ are contradictory indicators the smaller the assembly progress, the smaller δ_1_ is, which means the assembly system has a strong self-adjustment ability to prevent order delay anomalies and thus has a smaller chance to trigger inverse scheduling; while the smaller the assembly progress, the larger δ_2_ is, which means the adjustment space is larger, so inverse scheduling can avoid order delay due to insufficient adjustment space at the later stage of assembly. The difficulty of self-adjustment ability and adjustment space in triggering inverse scheduling is illustrated in Fig. 1. As the assembly schedule progresses, the self-adjustment ability decreases, making it more likely to trigger inverse scheduling. In contrast, the adjustment space becomes smaller and smaller as the assembly progresses; therefore, it becomes more difficult to trigger inverse scheduling.

In this paper, an adaptive tolerance driving mechanism is proposed, in which the self-adjustment ability indicator δ_1_ competes with the adjustment space indicator δ_2_. The adaptive tolerance δ is given by Eq. (13).

13 $$\delta = \max \left\{ {\frac{1}{{1 - \delta_{1} }},\delta_{2} } \right\} \cdot \frac{{C_{{\text{Delay time}}} }}{{C_{{\text{Total completion time}}} }}$$

Among Eq. (12), δ_1_ and δ_2_ are the self-adjustment ability and adjustment space, respectively, and δ is the delay tolerance allowed for aircraft flat-tail order delivery. The maximum value of the delay tolerance δ_max_ needs to be determined before the aircraft flat-tail assembly inverse scheduling.

The inverse scheduling based on adaptive tolerance driving mechanism can adopt an adaptive inverse scheduling driving strategy according to different assembly stages, which not only considers the self-adjustment ability of the assembly system, avoids frequent inverse scheduling, ensures the stability of assembly production, but also takes into account the effectiveness of scheduling, which enables the assembly system to respond to order delay abnormal events in a timely manner and ensures an on-time delivery level of the order.

## Hybrid genetic algorithm

The FFISP inverse scheduling model involves a large number of variables, and the assembly time for each process of each assembly task needs to be determined. The scale of the problem is enormous, making it difficult to solve directly by exact algorithms or mathematical planning methods. The genetic algorithm, as a swarm intelligence optimisation algorithm, possesses a strong global search capability and is well-suited for solving large-scale combinatorial optimisation problems. At the same time, to prevent the genetic algorithm from converging to a local optimum, a local search strategy is introduced to improve the genetic algorithm. Therefore, an improved genetic algorithm (IGA) based on the genetic algorithm and local search strategy is used to solve the FFISP problem in this paper.

### IGA* algorithm steps*

The specific steps of the IGA can be seen as follows:

Step 1: Initialise the parameters.

Initialise the parameters of the IGA algorithm, including population size *popsize*, crossover probability *Pr*, variation probability *Pc*, elite retention ratio *Pm*, and the maximum number of iterative generations *maxGen*.

Step 2: Determine the coding rules.

The FFISP problem contains two essential codes: one is the scheduling sequence code, and the other is the assembly time code. The scheduling sequence code and the assembly time code should correspond to each other. In FFISP problems, the scheduling sequence code should contain the assembly frame information, the assembly task information, and the corresponding process information. Meanwhile, the assembly time code should include the assembly time selected by the process in the corresponding scheduling sequence.

In response to the different requirements of the FFISP problem for scheduling sequence coding and assembly time coding, two different coding rules are adopted in designing the coding rules.

For the scheduling sequence coding, a fractional coding approach is employed to encode the scheduling sequence, imitating the coding approach of random keys proposed by Bean^29^. Taking the assembly frames *M*_*1*_~*M*_*m*_ as the sequence, the real number coding with decimal places is used. The integer part of each gene indicates the first few assembly tasks, and the decimal part indicates the subsequent processes of that assembly task. For example, suppose the coding sequence on assembly frame M1 is [4.2, 3.3, 5.4, 6.3, 2.5]. In that case, it means that process 2 of assembly task 4, process 3 of assembly task 3, process 4 of assembly task 5, process 3 of assembly task 6, and process 5 of assembly task 2 are processed on assembly frame *M*_*1*_ in that order. The scheduling sequence codes on assembly frames *M*_*1*_~*M*_*m*_ are combined to form the scheduling sequence codes of FFISP.

For the assembly time coding, an integer real number coding method is adopted, corresponding to the scheduling sequence. The value of each gene represents the assembly time of the process on the corresponding assembly frame for the assembly task corresponding to the scheduling sequence coding.

Step 3: Generate the initial population.

The initial population is the initial assembly frame time chromosome, which is generated randomly according to the initial solution *S* of the scheduling. In solving the inverse scheduling problem of aircraft flat-tail assembly production, the initial solution *S* is the solution to the static scheduling problem of aircraft flat-tail assembly production.

Step 4: Adaptation value evaluation.

The adaptation value evaluation is an assessment of the quality of individuals in a population, which requires calculating the adaptation value of each individual in the population first. The process is as follows:

(1) Calculate each objective function value *f*_*1*_*(x)* and *f*_*2*_*(x)* for each individual of the population.

(2) Objective function de-quantization. For multi-objective optimization, the standard value of the objective function is obtained by de-quantization^31^. The bi-objective de-quantization formula of FFISP is shown in Eqs. (14) and (15).

14 $$\mathop f\nolimits_{1}{\prime} = \frac{{f_{1} - f_{1} (best)}}{{f_{1} (worst) - f_{1} (best)}}$$

15 $$\mathop f\nolimits_{2}{\prime} = \frac{{f_{2} - f_{2} (best)}}{{f_{2} (worst) - f_{2} (best)}}$$

*f*_*1*_*(best)* and *f*_*1*_*(worst)* are the optimal and worst values of the objective function 1, respectively, and *f*_*2*_*(best)* and *f*_*2*_*(worst)* are the optimal and worst values of the objective function 2, respectively.

(3) Calculate the adaptation value *f* for each individual. According to the utility function method, assign different weights *w*_*1*_ and *w*_*2*_ to objective functions 1 and 2, respectively.

16 $$f = w_{1} \cdot \mathop f\nolimits_{1}{\prime} + w_{2} \cdot \mathop f\nolimits_{2}{\prime} .$$

Step 5: Select an elite retention strategy.

To evolve the optimisation direction of the genetic algorithm towards optimising the objective function, it is necessary to select the better individuals for retention and eliminate the poor ones in due course. In this paper, a roulette selection strategy^32^ is proposed, which is a genetic selection operation based on the size of the adaptation value of individuals in the population, determined by probability. Through the roulette selection strategy, individuals with better adaptation values have a greater probability of being passed on to the next generation, while ensuring that each individual has a certain probability of being retained. This approach provides both superiority and, to some extent, the diversity of the offspring population.

Step 6: The two points crossover.

Crossover is the process by which the genetic algorithm generates new child individuals through certain combinatorial operations. A good crossover strategy can perform an efficient search of the solution space while ensuring the desirable characteristics of the parent individuals, thereby determining the global search capability of the genetic algorithm. In this paper, a two-point crossover method is introduced.

**Fig. 2.**
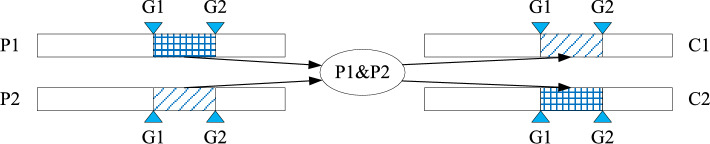
The two-point crossover operation.

Two parent individuals are randomly selected, designated as P1 and P2, and two genes, G1 and G2, with an interval distance greater than or equal to 1, are chosen randomly at the exact positions of P1 and P2. The chromosome sequences of parent individuals P1 and P2 are then exchanged between generations G1 and G2. The procedure of the two-point crossover operation is shown in Fig. 2.

Step 7: Mutation.

**Fig. 3.**
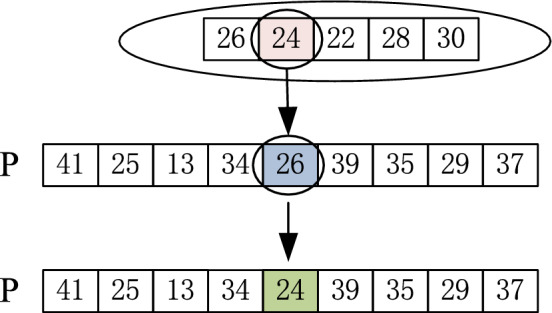
The mutation operation.

The variation is to maintain the population diversity and improve the quality of the solution. For the characteristics of assembly time coding, the assembly time of a process is represented by a series of discrete values, each occurring at a fixed interval. Therefore, a gene is randomly selected, and a specific assembly time from its candidate assembly time set is randomly selected for replacement. The mutation operation is shown in Fig. 3.

Step 8: Local search.

Combining the genetic algorithm and local search can leverage the global search capability of the genetic algorithm and the local search ability of local search methods.

The local search is performed by randomly selecting a gene location *i* with a probability of *P_ls* = 0.2. A neighbourhood search is conducted within this location by iterating over all possible assembly times in the candidate set *P_ijh* for that specific process. The size of this candidate set (typically 5–10 discrete values) defines the neighbourhood scope. The current individual is replaced with the one that yields the best solution in this local neighbourhood.

Step 9: *Gen*=*Gen*+1, determine whether the termination condition is satisfied; if so, output the optimal solution; if not, return to Step 4 and loop Step 4~Step 8.

**Fig. 4.**
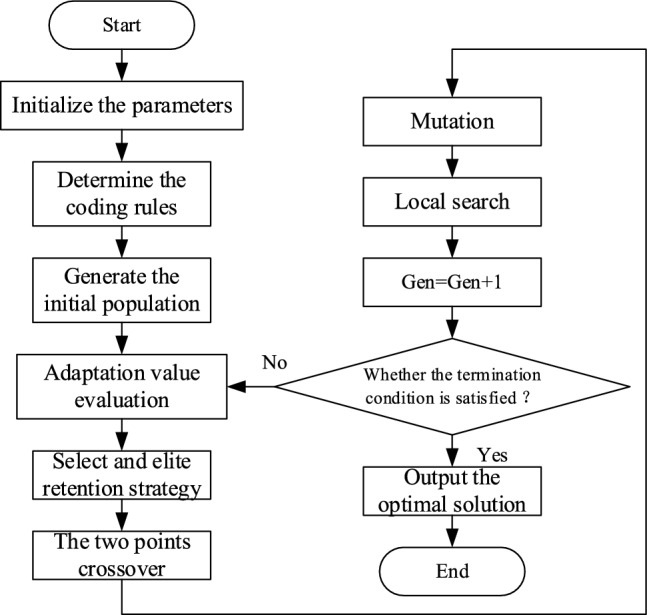
The improved genetic algorithm flow chart.

The flow chart of the improved genetic algorithm is shown in Fig. 4.

## Experimental results and analysis

### Evaluation indicators of inverse scheduling results

#### Evaluation indicators for the effectiveness of inverse scheduling results

The improvement in the objective function of the inverse scheduling relative to the pre-scheduled solution measures the effectiveness of the inverse scheduling approach. The objective function of inverse scheduling in this paper is to minimize the delay penalty cost and minimize the assembly time adjustment cost, and *Ee* is defined as the difference between the objective function of the inverse scheduling scheme and the pre-scheduling scheme, which is shown in the Eq. (17), and the larger *Ee* is, the more effective the optimization effect of the inverse scheduling scheme is.

${f}{\prime}$ is the objective function value of the inverse scheduling scheme, and *f* is the objective function value of the pre-scheduling scheme.

17 $$E_{e} = f - f{\prime}$$

#### Evaluation indicators of inverse scheduling stability

Stability is another important consideration for aircraft flat-tail assembly production. When adjusting the production schedule for aircraft flat-tail assembly, it is crucial to minimize significant adjustments to the assembly production schedule to maintain a balanced supply of in-house and outsourced parts. To minimize the time deviation between each assembly process of pre-scheduling and inverse-scheduling, thereby preserving the stability of aircraft flat-tail assembly production, stability indicators are introduced to evaluate the inverse-scheduling assembly plan. The stability indicator *E*_*S*_ is calculated by summing the deviations of the start assembly times of the processes that have not yet been assembled in both pre-scheduled and inverse-scheduled scenarios, as shown in Eq. (18).

18 $$E_{s} = \min \sum\limits_{j = 1}^{n} {\sum\limits_{h = 1}^{n^{\prime}j} {\left( {\left| {s_{jh} ^{\prime} - s_{jh} } \right|} \right)} }$$

*S*_*jh*_ indicates the start time of the *h*^*th*^ process of assembly task *j* in the pre-scheduling, *S*_*jh*_^***’***^ indicates the start time of the *h*^*th*^ process of assembly task *j* in the inverse scheduling scheme, and *n*_*j*_^***’***^ means the total number of processes of assembly task *j* in the pre-scheduling scheme that have not been completed at the time of inverse scheduling.

### Performance verification of the self-adaptive tolerance driving mechanism

To verify the effectiveness and superiority of the inverse scheduling strategy based on the self-adaptive tolerance driving mechanism in solving the dynamic scheduling of aircraft flat-tail assembly production, this paper compares and experimentally validates four inverse scheduling strategies, namely, self-adaptive tolerance driving mechanism (SAD), event driving mechanism (ED), period driving mechanism (PD), and delivery tolerance driving mechanism (DTD) mentioned in the literature^18^.

The data of the test cases are shown in Table 2. The algorithm is programmed in C#, and the hardware environment consists of an AMD Ryzen 7 6800H processor (3.20 GHz) with 32 GB of memory and a 64-bit Windows 11 operating system.

**Table 2:** Performance test cases for the inverse scheduling strategy.

Parameters	Range of values
Number of assembly tasks	12
Number of work processes	12
Assembly time	U[20,60]
Number of assembly frames	U[2,6]
Delivery time for assembly tasks	U[1200,1800]
Order delay penalty ($1000/hour)	U[2,5]

The solution results are significantly affected by the parameter settings of genetic algorithms. To efficiently select the parameter combinations for the IGA algorithm, this paper employed orthogonal experimental design (OED) to test multiple parameter combinations. Through parameter adjustments, five important parameters and their corresponding ranges that significantly impact the experimental results were identified. A 3-factor, 3-level experimental design table (as shown in Table 3.) was designed to optimize parameters such as population size (*popsize*), crossover probability (*Pc*), mutation probability (*Pm*), and maximum iterations (*maxGen*). A L9(3^4) mixed orthogonal table (as shown in Table 4.) was used to test 3 levels for each parameter. The 6X8 example was selected as the test object, with average objective function values calculated for each combination (Table 5.).

**Table 3:** Range of key parameters for medium problem size.

Experiment No.	Population size *popsize*	Crossover probability *Pc*	Mutation probability *Pm*
1	50	0.7	0.05
2	100	0.8	0.1
3	150	0.9	0.15

**Table 4:** L9(3^4^) Orthogonal table for parameter optimization.

Experiment No.	Population size *popsize*	Crossover probability *Pc*	Mutation probability *Pm*	*maxGen*
1	50	0.7	0.05	100
2	50	0.8	0.1	200
3	50	0.9	0.9	300
4	100	0.7	0.1	300
5	100	0.8	0.15	100
6	100	0.9	0.05	200
7	150	0.7	0.15	200
8	150	0.8	0.05	300
9	150	0.9	0.1	100

**Table 5:** Orthogonal experiment results (6X8 Example).

Experiment no.	Objective function value (*f*)	Range (R)	Optimal level
1	138.6	-	-
2	132.1	-	-
3	129.5	-	-
4	127.8	-	-
5	126.4	-	-
6	125.3	8.2	*popsize*=100
7	130.2	6.5	*Pc*=0.8
8	131.7	5.9	*Pm*=0.1
9	133.9	7.3	*maxGen*=200

Range analysis showed the optimal parameter combination: *popsize*=100, *Pc*=0.8, *Pm*=0.1, *maxGen*=200, with *f*=125.3 (11.4% lower than the worst combination). The improved genetic algorithm designed in the previous section is applied to all four inverse scheduling strategies.

**Fig. 5:**
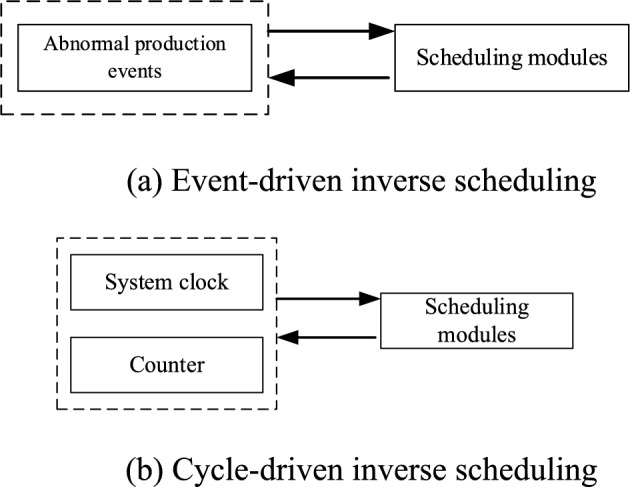
(**a**) Event-driven inverse scheduling (**b**) Cycle-driven inverse scheduling.

The event driving mechanism (ED) means that once an abnormal production event occurs in the system, inverse scheduling will be performed immediately, and its scheduling model is shown in Fig. 5(a); the period driving mechanism (PD) means that the system performs inverse scheduling once every period or times, which is mainly controlled by the system clock or counter, and its scheduling model is shown in Fig. 5(b); the delivery tolerance driving mechanism (DTD) is essentially an event driving rolling scheduling method, which minimises unnecessary inverse scheduling by setting a certain delivery deviation tolerance; the self-adaptive tolerance driving (SAD) is also essentially an event driving mechanism, which taking into account the self-adjustment ability and adjustment space of different assembly stages. To compare the effectiveness of the above four inverse scheduling driving mechanisms, the inverse scheduling period T of the cycle driving mechanism, the deviation tolerance of the delivery time deviation tolerance driving mechanism δ_DTD_ and the deviation tolerance of the self-adaptive tolerance driving mechanism δ_SAD_ should be determined in advance.

For the inverse scheduling period T of the cycle driving mechanism and the deviation tolerance of the delivery deviation tolerance driving mechanism δ_DTD_, the inverse scheduling period T=100 of the cycle driving mechanism and the deviation tolerance δ_DTD_=0.115 of the delivery deviation tolerance driving mechanism are determined according to the method of determining the delivery deviation tolerance and the size of the rolling window proposed in the literature^8^, combined with the data of the aircraft flat-tail assembly production inverse scheduling strategy test cases in Table 2.

To determine the deviation tolerance of the self-adaptive tolerance driving mechanism, several experiments using the test arithmetic data in Table I are conducted, and the objective function value *f* and the stability indicator *E*_*S*_ are recorded. The experimental results are shown in Table 6.

**Table 6:** Results of the self-adaptive tolerance deviation experiment.

Self-adaptive tolerance deviation	Target optimisation the function value (*f*)	Number of inverse scheduling times	Stability value (*E*_*S*_)
0.0025	318.5	17	1390
0.005	312.4	14	1230
0.0075	310.2	11	1150
0.0125	301.3	8	1020
0.025	288.1	7	920
0.05	276.8	5	830
0.075	287.2	3	850
0.10	296.7	2	940
0.125	303.3	2	1010
0.15	306.2	1	1190
0.175	308.3	1	1240
0.20	312.1	0	1380
0.225	314.3	0	1470
0.25	317.2	0	1550

**Fig. 6:**
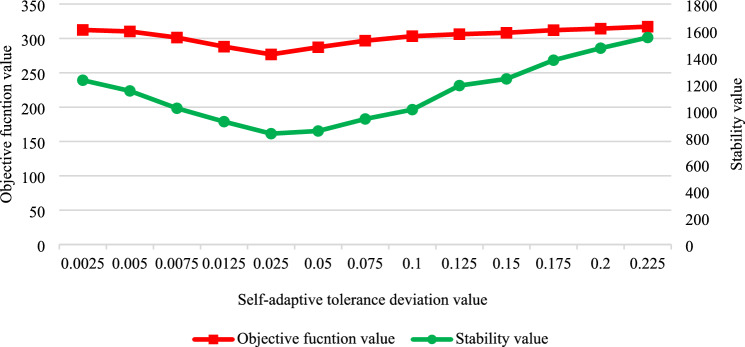
Results analysis of the self-adaptive tolerance deviation experiment.

The data in Table 6 is visualised in Fig. 6, where the horizontal axis represents the value of the self-adaptive tolerance deviation, and the left and right vertical axes represent the value of the objective function value and the stability value, respectively. It can be observed that, as the self-adaptive tolerance deviation increases gradually, both the objective function value and the stability value exhibit a concave curve. The reason is that: (1) when the self-adaptive tolerance deviation is slight, the inverse scheduling is more frequent, and once there is an abnormal event in the assembly production schedule, the inverse scheduling is easily triggered, and the frequent inverse scheduling not only leads to the poor smoothness of the assembly production, but also leads to the fact that the assembly time self-regulation ability in the assembly production process cannot be fully utilized, and the objective of the inverse scheduling of the assembly production is not well optimized; (2) when the self-adaptive tolerance deviation is significant, the number of inverse scheduling is small because the threshold value of inverse scheduling trigger is higher, and thus the abnormal events of assembly progress cannot be handled in time, which leads to a larger deviation of the actual execution of the assembly plan compared with the pre-scheduling scheme, and thus the objective function value and stability value are poor. As shown in Fig. 6, the objective function value and stability value are more optimal when *δ*_*SAD*_ is set to 0.05.

To compare the performance of the four inverse scheduling driving mechanisms, simulation experiments are conducted by using the nine sets of arithmetic cases in Table 7, and the computational results are shown in Tables 8 and 9.

**Table 7:** Arithmetic cases.

Case number	Experimental group	Number of tasks	Number of processes	Assembly time	Number of frames	Delivery Period	Delay penalty
1	6X4	6	4	U[20,60]	U[2,6]	U[100,200]	U[2,5]
2	6X8	6	8	U[20,60]	U[2,6]	U[400,800]	U[2,5]
3	6X12	6	12	U[20,60]	U[2,6]	U[800,1200]	U[2,5]
4	12X4	12	4	U[20,60]	U[2,6]	U[300,600]	U[2,5]
5	12X8	12	8	U[20,60]	U[2,6]	U[600,1000]	U[2,5]
6	12X12	12	12	U[20,60]	U[2,6]	U[1200,1800]	U[2,5]
7	30X4	30	4	U[20,60]	U[2,6]	U[600,900]	U[2,5]
8	30X8	30	8	U[20,60]	U[2,6]	U[900,1200]	U[2,5]
9	30X12	30	12	U[20,60]	U[2,6]	U[1600,2400]	U[2,5]

**Table 8:** Simulation results of inverse scheduling driving mechanism - objective function value.

Experimental group	Objective function value (*f*)							
	SAD		ED		PD		DTD	
	*min*	*avg*	*min*	*avg*	*min*	*avg*	*min*	*avg*
6X4	34.2	37.1	45.2	49.7	40.3	45.6	32.8	38.3
6X8	119.2	127.9	129.2	137.7	126.3	134.4	125.1	132.1
6X12	110.3	115.2	117.4	125.2	114.3	122.9	112.3	120.6
12X4	136.3	143.2	157.3	165.9	152.4	163.8	147.5	156.2
12X8	248.9	254.2	266.4	276.8	270.1	277.3	253.3	260.5
12X12	272.2	276.3	290.1	295.3	292.5	296.2	283.4	288.5
30X4	182.0	188.3	201.2	214.1	207.6	215.8	197.5	205.6
30X8	293.2	301.8	304.5	321.4	319.7	330.9	302.5	317.8
30X12	319.0	323.5	334.5	350.1	342.3	357.5	332.7	347.7

**Table 9:** Simulation results of the inverse scheduling driving mechanism - stability value.

Experimental group	Stability value (*E*_*S*_)							
	SAD		ED		PD		DTD	
	*min*	*avg*	*min*	*avg*	*min*	*avg*	*min*	*avg*
6X4	273	278	283	289	279	285	282	287
6X8	382	391	388	399	385	393	382	402
6X12	439	452	453	466	442	453	445	463
12X4	563	582	581	593	579	585	587	597
12X8	765	784	810	823	775	798	794	816
12X12	830	853	869	892	837	879	872	885
30X4	1086	1123	1142	1164	1105	1133	1132	1156
30X8	1235	1256	1278	1311	1249	1277	1271	1299
30X12	1387	1403	1433	1479	1412	1439	1425	1458

In terms of the optimisation effectiveness of the objective function, it can be seen from Table 8 that the solution effectiveness of SAD is significantly better than the other three driving mechanisms, followed by DTD. In contrast, the solution quality of ED and PD is relatively poor. For the small-scale inverse scheduling problem, the solution quality of PD is better than that of ED, while the solution quality of ED gradually improves over that of PD as the problem size increases. At the same time, the solution quality of SAD is significantly better than that of the other three driving mechanisms, regardless of the solution size.

In terms of the optimization effectiveness of the objective function, it can be seen from Table 8 that the solution effectiveness of SAD is significantly better than the other three driving mechanisms, followed by DTD, while the solution quality of ED and PD is relatively poor. For the small-scale inverse scheduling problem, the solution quality of PD is better than that of ED, while the solution quality of ED is gradually better than that of PD as the problem size increases, while the solution quality of SAD is significantly better than that of the other three driving mechanisms regardless of the solution size.

In terms of stability indicators, Table 9 shows that SAD is significantly more stable than the other four driving mechanisms, followed by the cyclic driving mechanism PD. The event-driven mechanism (ED) and the delivery deviation tolerance mechanism (DTD) are less stable, and their optimization effects are comparable. For the dynamic scheduling of aircraft flat-tail assembly, which has a long assembly cycle and frequent abnormal events, both ED and DTD will lead to more frequent reverse scheduling. Therefore, their production stability is inferior to PD. The most suitable inverse scheduling is considered to ensure the optimal solution result.

**Fig. 7:**
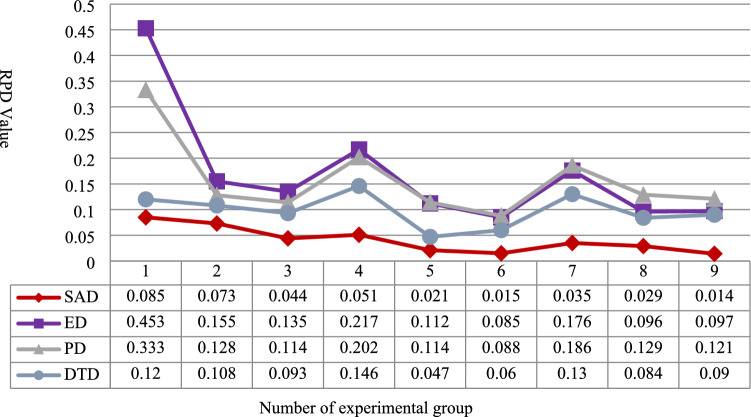
Comparison of RPD of objective function values.

**Fig. 8:**
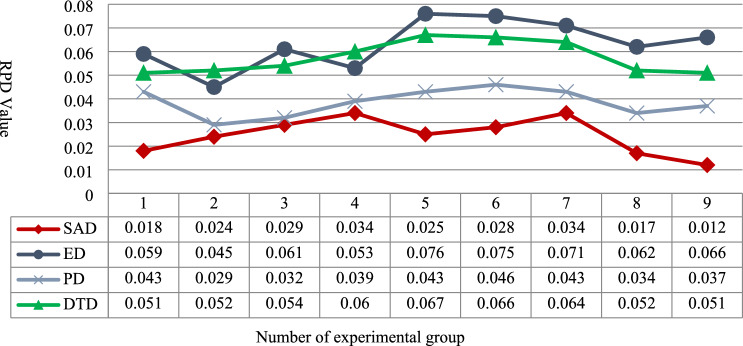
Comparison of RPD of stability values.

To visually compare the solution optimisation results of each algorithm, the data in Table IV and Table V are processed by using relative percentage deviation, and the RPD effectiveness of the four inverse scheduling driving mechanisms are compared as shown in Fig. 7 and Fig. 8 respectively, with both horizontal coordinates indicating the experimental group numbers and both vertical coordinates indicating the RPD values. It can be seen that the SAD self-adaptive tolerance driving mechanism outperforms the other three driving mechanisms, both in terms of objective function optimisation and assembly production stability.

For the inverse scheduling of aircraft flat-tail assembly production, the number of inverse scheduling and the time point of inverse scheduling have a significant influence on the production of aircraft flat-tail assembly. In the actual assembly production process, it is crucial to reduce the number of inverse scheduling and improve the efficiency of inverse scheduling as much as possible to improve the on-time delivery ability of aircraft flat-tail and increase the utilization rate of the assembly frame. For this reason, the number of inverse scheduling and the time point of inverse scheduling will be compared to the solution effectiveness of the four driving mechanisms, and the nine sets of cases in Table 3 will be used as the test cases. For the inverse scheduling time point, to facilitate the vertical comparison of cases of different sizes, for each case, the minimized maximum completion time is taken as the maximum scheduling period, and it is divided into three equal parts, which are called pre-scheduling, mid-scheduling and post-scheduling, and the number of times each inverse scheduling falls within each scheduling period is counted. The calculation results are shown in Table 10.

**Table 10:** Simulation experiment results-inverse scheduling times and time distribution.

Experimental group	Inverse scheduling times and time distribution															
	SAD				ED				PD				DTD			
	Pre	Mid	Post	Total	Pre	Mid	Post	Total	Pre	Mid	Post	Total	Pre	Mid	Post	Total
1	0.6	0.5	0.1	1.2	1.2	0.4	1.1	2.7	0.6	0.6	0.8	2	0.3	0.7	0.4	1.4
2	0.7	0.9	0.2	1.8	0.7	2.4	1.3	4.4	1	1	1	3	0.4	0.9	0.8	2.1
3	0.8	1.3	0.2	2.3	2.1	1.6	2.6	6.3	1.6	1.6	1.8	5	0.3	1.2	0.9	2.4
4	1.3	1.6	0.3	3.2	3.4	2.2	3.0	8.6	2	2	2	6	0.6	1.7	1.3	3.6
5	1.9	2.4	0.4	4.7	2.0	3.8	3.4	9.2	2.3	2.3	2.4	7	0.9	2.5	1.8	5.2
6	1.8	3.0	0.4	5.2	1.8	4.1	3.5	9.4	2.3	2.3	2.4	7	0.8	2.9	2.0	5.7
7	1.7	3.5	0.7	5.9	4.2	3.7	2.8	10.7	2.6	2.6	2.8	8	1.1	3.2	3.0	6.3
8	2.1	3.7	0.5	6.3	5.3	4.2	1.8	11.3	2.6	2.6	2.8	8	1.2	3.1	3.3	6.6
9	1.8	4.2	0.4	6.4	3.2	5.6	3.4	12.2	3	3	3	9	1.1	3.3	3.5	6.9

As can be seen from Table 10, the number of inverse scheduling for SAD is significantly less than that of ED, PD, and DTD, and the number of inverse scheduling for each inverse scheduling driving mechanism is SAD < DTD < PD < ED. From the viewpoint of the time distribution of inverse scheduling, the inverse scheduling of SAD is mainly concentrated in the early and middle stages, because the self-regulating ability and adjustment space of assembly production are larger in the early and middle stages of assembly. Thus, the inverse scheduling in the early and middle stages can achieve better results. The inverse scheduling of ED occurs the most frequently, and its time distribution is more random. This is because the trigger for inverse scheduling, based on the event-driven mechanism, is related to abnormal events in assembly production. Consequently, the assembly production environment influences the frequency of inverse scheduling. PD is a fixed-cycle inverse scheduling, so the number of inverse scheduling and the distribution of events are related to the execution time of the assembly production. The inverse scheduling of DTD is mainly concentrated in the middle and late stages, because in the early stage of assembly, the tolerance of deviation from the delivery date is greater. In contrast, the abnormal events of assembly production in the middle and late stages of assembly are more likely to lead to delays in order delivery; thus, the number of inverse scheduling instances is more frequent, and the distribution is more intensive.

Through the comparative simulation experiments mentioned above, it can be seen that the inverse scheduling strategy based on the self-adaptive tolerance driving mechanism outperforms the other three inverse scheduling strategies in terms of the optimisation quality of the objective function, the inverse scheduling stability indicator, and the number of inverse scheduling times, thus proving the effectiveness and superiority of the inverse scheduling strategy based on the self-adaptive tolerance driving mechanism in solving the dynamic scheduling of aircraft flat-tail assembly production.

### Performance comparison of algorithms

To further validate the effectiveness of the proposed Improved Genetic Algorithm (IGA), we compared it against several established algorithms: a Standard Genetic Algorithm (SGA) without the local search component, the well-known Non-dominated Sorting Genetic Algorithm II (NSGA-II)^33^, and the Multi-objective Evolutionary Algorithm based on Decomposition (MOEA/D)^34,35^. All algorithms were implemented with the same population size and maximum generation count for fairness. Performance was evaluated using the Hypervolume (HV) and Inverted Generational Distance (IGD) metrics, which comprehensively measure convergence and diversity.

Results in Table 11. show that IGA consistently achieves higher HV and lower IGD values across different problem scales, confirmig its superior ability to find a diverse and well-converged set of Pareto-optimal solutions. The significant outperformance over SGA highlights the crucial role of the local search operator. While NSGA-II and MOEA/D are competitive, IGA maintains a slight but consistent edge, particularly in larger instances, demonstrating its robustness and effectiveness for the specific FFISP.

**Table 11:** Comparison of algorithm performance (Average HV/IGD over 10 runs).

Experimental group	IGA (Proposed)	SGA	NSGA-II	MOEA/D
6X4	0.752/15.3	0.681/24.7	0.738/16.8	0.745/15.9
12X8	0.698/28.1	0.602/45.6	0.674/30.4	0.685/29.2
30X12	0.641/42.5	0.523/68.9	0.618/45.1	0.625/43.8

### Performance verification of inverse scheduling method

For dynamic events in the production process, the most commonly used dynamic scheduling method is the rescheduling method, which reasonably adjusts the scheduling sequence, allowing the production plan to achieve a better scheduling result. However, for complex product manufacturing systems such as aircraft flat-tail, the pre-determined production plan often cannot be adjusted to a large extent due to the multi-departmental collaboration, and changing the production sequence of the original production plan will incur large production plan change costs, and adjusting the assembly plan sequence will incur high material handling costs and take up long material handling time and flat-tail frame replacement time and fixed clamping time. For this reason, the inverse scheduling method of aircraft flat-tail is proposed in this paper. To verify the effectiveness and superiority of the inverse scheduling method, this section will compare the solution effectiveness of the inverse scheduling method and the rescheduling method through an arithmetic test.

In the comparison method, the inverse scheduling method is solved by the improved genetic algorithm (IGA), and the inverse scheduling driving mechanism adopts the inverse scheduling strategy based on self-adaptive tolerance. At the same time, the rescheduling method (RM) will utilise the improved ant colony algorithm proposed in the literature^8^, and the inverse scheduling driving mechanism also employs an inverse scheduling strategy based on self-adaptive tolerance.

Since the material handling cost is much higher than the adjustment cost of processing workers in the actual assembly process of the aircraft flat-tail, the indicators of the cost objective function are not realistic, therefore, the performance comparison of the inverse scheduling method and the rescheduling method will be compared mainly from two perspectives of minimising the delay time and stability indicators. The test cases still utilise the nine sets of cases in Table III and add the wage level data for assembly workers: the wage level is 50 yuan per hour when the assembly workers are working normally. The wage level is doubled, that is, 100 yuan per hour, when workers are added dynamically at each assembly station.

The solution results of the inverse scheduling method and the rescheduling method are shown in Table 12.

**Table 12:** Results of the inverse scheduling method validation algorithm.

Experimental group	Total delay time (/h)				Stability value (*E*_*S*_)			
	IS		RM		IS		RM	
	*min*	*avg*	*min*	*avg*	*min*	*avg*	*min*	*avg*
6X4	11	12.4	13	15.4	273	278	285	293
6X8	40	42.6	45	48.2	382	391	421	437
6X12	37	38.4	43	45.8	439	452	486	498
12X4	45	47.7	52	57.6	563	582	630	647
12X8	83	84.7	86	90.3	765	784	833	845
12X12	91	92.1	94	97.9	830	853	910	923
30X4	61	62.8	65	68.3	1086	1123	1156	1164
30X8	98	100.6	102	106.8	1235	1256	1398	1409
30X12	106	107.8	110	114.6	1387	1403	1470	1482

From analysing the solution results, it can be seen that: (1) from the viewpoint of objective function optimisation, the solution results of IS are significantly better than RM, the reason is that the inverse scheduling method can flexibly increase part of the production resources, and thus its optimisation adjustment magnitude is significantly superior to that of the rescheduling method; (2) from the viewpoint of assembly production scheduling stability, the production stability of IS are much better than that of RM, the reason is that the rescheduling method is to adjust the scheduling sequence of all processes that have not yet started assembly, and thus its adjustment magnitude is larger. The reason is that the rescheduling method adjusts the scheduling sequence for all processes that have not yet started assembly, thus its adjustment magnitude is larger, and the stability of aircraft flat-tail assembly is poor.

Therefore, the inverse scheduling method is significantly superior to the rescheduling method in solving the dynamic scheduling problem of aircraft flat-tail assembly production in terms of the optimization quality of the objective function, the stability of aircraft flat-tail assembly production, and the production cost.

### Sensitivity analysis

A sensitivity analysis was conducted to assess the impact of key parameters on the algorithm’s performance. Firstly, we varied the weight coefficients (*w*_*1*_, *w*_*2*_) in the utility function (Eq. 16). As expected, higher *w*_*1*_ prioritized solutions with lower delay costs, while higher *w*_*2*_ favored solutions with lower adjustment costs. The IGA proved robust, successfully shifting the search focus according to the specified weights without significant degradation in solution quality for either objective.

Secondly, we analyzed the sensitivity of the self-adaptive tolerance driving mechanism to the maximum tolerance parameter *δ*_*max*_. Setting *δ*_*max*_ too low (e.g., 0.01) led to frequent and unnecessary inverse scheduling, increasing computational overhead without significant benefit. Setting it too high (e.g., 0.3) caused delayed responses to disruptions, leading to higher overall delay costs. The value of *δ*_*max*_ =0.05, determined earlier, was confirmed to be a robust setting that effectively balances reactivity and stability.

## Enterprise case validation

**Fig. 9:**
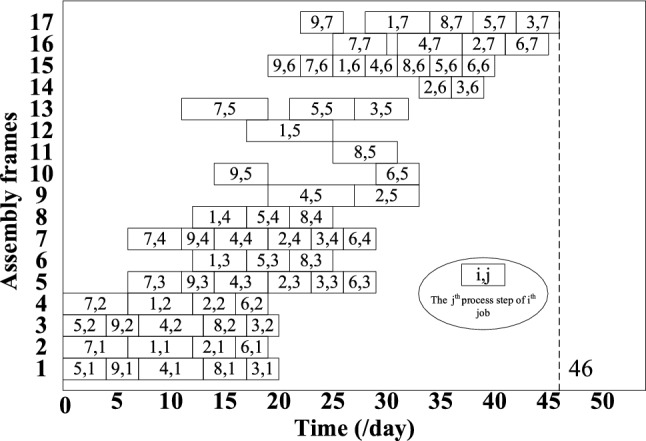
Gantt chart without inverse scheduling.

**Fig. 10:**
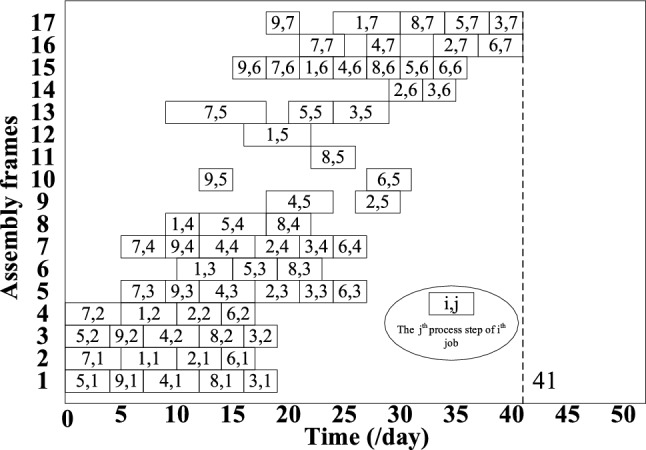
Gantt chart with inverse scheduling.

To verify the effectiveness and superiority of the inverse scheduling strategy and hybrid genetic algorithm based on the self-adaptive tolerance driving mechanism proposed in this paper, this section uses actual data from a type of aircraft flat-tail at an aircraft manufacturing plant in Shanghai for example verification. By verifying its actual production data, the Gantt charts before and after inverse scheduling are shown in Fig. 9 and Fig. 10, respectively. The order delay penalty costs are $27,920 and $10,450, respectively, and the worker adjustment cost is $6,500. The inverse scheduling strategy and hybrid genetic algorithm proposed in this paper, based on a self-adaptive tolerance driving mechanism, can effectively shorten the flat-tail assembly cycle, reduce order delay costs, and enhance flat-tail delivery capability.

## Conclusion

This paper examines the problem of inverse scheduling in the production of aircraft flat-tail assemblies. To address this problem, a model for the flexible flow-shop inverse scheduling problem (FFISP) in aircraft flat-tail assembly production was proposed. We then develop an improved genetic algorithm (IGA) to solve the FFISP, which includes a coding scheme, crossover scheme, mutation scheme, and variable neighbourhood search. Additionally, we designed an inverse scheduling strategy based on a self-adaptive tolerance driving mechanism. The effectiveness of the self-adaptive tolerance driving mechanism and the IGA algorithm is verified using different scales, and the IGA algorithm is compared with several efficient algorithms. Our results confirm that the inverse scheduling method outperforms the rescheduling method in terms of optimisation quality of the objective function, assembly production stability, and production cost. A case study conducted in an enterprise demonstrates the effectiveness of the proposed inverse scheduling method for flat-tail assembly production, particularly in handling abnormal events.

The proposed method provides a practical decision-support tool for production managers in aviation assembly shops, enhancing on-time delivery capabilities and reducing penalty costs. For future work, we plan to integrate the inverse scheduling framework with Industry 4.0 technologies such as digital twins for real-time synchronization between physical and virtual assembly shops, and large language models for intelligent interpretation and automatic handling of exception events. Furthermore, extending the application to other complex manufacturing domains, such as shipbuilding and energy turbine assembly, represents a promising research direction. Moving forward, our research will focus on addressing more realistic inverse scheduling problems that consider additional parameters and constraints in the aircraft flat-tail assembly workshop. Additionally, exploring and designing new and efficient intelligent algorithms will be a crucial aspect of our future work.

## Data Availability

The data that support the findings of this study are included in this article, and also available from the corresponding author upon reasonable request.
